# Treatment of Epistaxis by Insufflations of Alum

**Published:** 1847-09

**Authors:** 


*Treatment of Epistaxis by Insufflations of Alum.*—When hemorrhage from the nasal cavities assumes a dangerous aspect, recourse is generally had to plugging, a measure both inconvenient and painful. M. Lecluyse has successfully employed means far more simple, and at the same time, according to his own account, more certain—namely, the insufflation by means of a quill of equal parts of powdered gum arabic and alum. In one case this succeded after three repetitions; other means, and plugging among them, having entirely failed.—*Prov. Med. Jour.*—*Ibid.*

